# IFIT3 (interferon induced protein with tetratricopeptide repeats 3) modulates STAT1 expression in small extracellular vesicles

**DOI:** 10.1042/BCJ20210580

**Published:** 2021-11-09

**Authors:** Nicole M. Naranjo, Israa Salem, Maisha A. Harris, Lucia R. Languino

**Affiliations:** 1 Prostate Cancer Discovery and Development Program, Thomas Jefferson University, Philadelphia, PA 19107, U.S.A; 2 Department of Cancer Biology, Sidney Kimmel Cancer Center, Thomas Jefferson University, Philadelphia, PA 19107, U.S.A.

**Keywords:** αvβ6 integrin, IFIT3, interferon-stimulated genes, prostate cancer, small extracellular vesicles, STAT1

## Abstract

We have previously shown that the αvβ6 integrin plays a key role in promoting prostate cancer (PrCa) and it can be transferred to recipient cells via small extracellular vesicles (sEVs). Furthermore, we have reported in a proteomic analysis that αvβ6 integrin down-regulation increases the expression of IFIT3 (interferon induced protein with tetratricopeptide repeats 3) in PrCa cells and their derived sEVs. IFIT3 is a protein well known for being an antiviral effector, but recently its role in cancer has also been elucidated. To study the relationship between IFIT3 and STAT1 (signal transducer and activator of transcription 1), an upstream regulator of IFIT3, in PrCa cells and their released sEVs, we used CRISPR/Cas9 techniques to down-regulate the expression of the β6 integrin subunit, IFIT3 or STAT1. Our results show that IFIT3 and STAT1 are highly expressed in PrCa cells devoid of the β6 integrin subunit. However, IFIT3 but not STAT1, is present in sEVs derived from PrCa cells lacking the β6 integrin subunit. We demonstrate that loss of IFIT3 generates sEVs enriched in STAT1 but reduces the levels of STAT1 in the cells. As expected, IFIT3 is not detectable in STAT1 negative cells or sEVs. We thus propose that the observed STAT1 enrichment in sEVs is a compensatory mechanism for the loss of IFIT3. Overall, these results provide new insights into the intrinsic role of IFIT3 as a regulator of STAT1 expression in sEVs and in intercellular communication in PrCa.

## Introduction

Prostate cancer (PrCa) is the most common cancer affecting men in the United States [1]. The occurrence of PrCa has been attributed to the deregulation of various signaling pathways, including the androgen receptor (AR) signaling pathway [2,3]. Of clinical significance, depleting androgen in PrCa cells causes regression of the prostate tumor [4]. However, androgen deprivation can lead to the development of a metastatic, androgen-independent phenotype for which no efficient therapy has yet been developed.

Besides androgen signaling, integrin signaling cascades are also deregulated in PrCa [5,6]. Integrins are transmembrane receptors that drive the related processes of cell survival, adhesion, and proliferation [7,8]. Many studies have shown that aberrations in integrin signaling are capable of driving tumor metastasis [8,9] and can lead to advanced PrCa metastasis [5]. In particular, the αvβ6 integrin, which is not detectable in healthy human and mouse tissues [10,11], is associated with metastatic phenotypes and poor survival in various malignancies, including breast [12], cervical [13], and colorectal [14] cancers. The αvβ6 integrin acts as a receptor for extracellular matrix proteins, where it can bind to different ligands, such as fibronectin, tenascin-C, LAP-TGF-β and vitronectin, all which have been implicated in mediating cell adhesion and migration *in vitro* [15]. Our laboratory, as well as other groups, have shown that the αvβ6 integrin plays a key role in promoting tumor growth *in vivo* [10,12,16].

We had also previously published that the αvβ6 integrin is enriched in small extracellular vesicles (sEVs) derived from PrCa cells [17]. sEVs are vesicles of endosomal origin with a size range of 50–200 nm [18,19]. sEVs are present in most biological fluids including blood, urine, and milk as well as cell culture medium [19]. Proteins that are enriched in sEVs include members of the tetraspanin family (CD9, CD63, and CD81), and the ESCRT family (TSG101 and Alix) [19]. sEVs have emerged as significant mediators of intercellular communications [20,21], playing a role in reprogramming the tumor microenvironment (TME) [22], modulating tumor cell activity [20] as well as promoting metastatic niche formation [23]. Furthermore, studies from our group have shown that sEVs derived from PrCa cells and containing integrins can functionally modulate non-cancerous cells within the prostate TME [16,24–26].

Our published proteomic analysis showed that αvβ6 integrin down-regulation causes increased expression of the signal transducer and activator of transcription 1 (STAT1) [24] and members of the interferon-stimulated genes (ISG), which encode proteins such as IFIT1, IFIT3, OASL, and MX2 [27,28] in PrCa cells and their released sEVs [24].

As a member of the STAT family, STAT1 mediates distinct cell functions and regulates transcription [29] by transducing signals from transmembrane receptors to their nuclear targets [30]. STAT1 has a well-defined role as being a tumor suppressor for different cancers [31,32]. In PrCa, specifically, it has been shown that loss of STAT1 results in increased cell growth and survival. Moreover, in AR-negative patients, it was demonstrated that loss of STAT1 increases cell viability, thereby promoting PrCa progression [33]. STAT1 is activated by type I interferon (IFN) stimulation, and it is a key protein in the JAK-STAT pathway [34]. In this pathway, STAT1 forms a heterotrimeric transcriptional complex with STAT2 in association with IFN regulatory factor 9 (IRF9) [27,34,35]. This transcriptional complex is termed ISG factor 3 (ISGF3) [27,28,35]. The ISGF3 complex translocates to the nucleus where it activates the transcription of ISG [28,35]. ISG play an essential role in controlling virus infections as they can reinforce the IFN response [28].

The ISG, like the interferon-induced proteins with tetratricopeptide repeats (IFITs) [28], are localized in the cytoplasm [35–37]; they are strongly induced by type I IFN and have been well-studied for their antiviral activity [35,38,39]. Specifically, IFIT3, also known as ISG60, IFIT4 [35,38], or retinoic acid inducible gene (RIG-G) [40], was first identified in the acute promyelocytic leukemia cell line NB4 [36]. In this study, NB4 treatment with all-trans retinoic acid (ATRA) or IFN α dramatically induced IFIT3 expression. Moreover, IFIT3 has been studied for its antiproliferative activity in different types of solid tumors, including, hepatocellular carcinoma [41] and lung cancer [42]. Notwithstanding, little is known regarding IFIT3 expression or its potential role in sEVs derived from cancer cells.

Even though it is well established that STAT1, as a component of the ISGF3 complex, binds to the IFIT promoters [38], little is known about STAT1 and IFIT3 regulation in PrCa cells and their released sEVs. Therefore, we investigated the role of IFIT3 in modulating STAT1 expression in PrCa cell-derived sEVs. Our findings demonstrate for the first time that IFIT3 regulates STAT1 expression in PrCa-derived cells and their derived sEVs. Overall, this study sheds light on the previously unappreciated role of IFIT3 in regulating sEV protein content in PrCa cells and in intercellular communication in cancer progression.

## Materials and methods

### Cells and culture conditions

PC3 PrCa cells were cultured in RPMI media with l-glutamine (Corning, U.S.A.) supplemented with 10% fetal bovine serum (FBS), and 1% penicillin/streptomycin (Corning Cellgro, U.S.A.) in a humidified atmosphere of 5% CO_2_ at 37°C.

Cells were transiently transfected using oligofectamine with 30 nM of non-silencing (NS) siRNA (Cat. No. D-001810-01-20; Dharmacon) or β6 (D2) siRNA duplex (IDT Incorporated) as previously described [43]. Transient down-regulation of STAT1 mRNA expression in PC3 wild-type (PC3-WT) cells was achieved using targeting human STAT1 (6) siRNA duplex (IDT Incorporated). The sequences of STAT1 siRNA are hs.Ri. STAT1.13.6, forward 5′-GACAUCAUUCGCAAUUACAAAGUCA-3′, reverse 5′-UGACUUUGUAAUUGCGAAUGAUGUCAG-3′.

Genomic depletion of the β6 integrin subunit in PC3-WT cells was performed as previously described [16]. The β6 integrin subunit genomic depletion resulted in PC3-β6KO CRISPR clones referred to as β6KO C5, and β6KO C7.

For genomic depletion of STAT1, IFIT3 and ITGB3 (β3 integrin subunit) the process was as follows: for *STAT1*, two gRNAs were designed to induce a frame-shift excision in the coding region of *STAT1* (gRNA1: 5′-GGTGGCAAATGAAACATCAT, gRNA2: 5′-GTGACAGGAGGTCATGAAAA) (Synthego Corporation, Redwood City, CA). For genomic depletion of *IFIT3*, two gRNAs were designed to induce a frame-shift excision in the coding region of *IFIT3* (gRNA1: 5′-TGAGGTCACCAAGAATTCCC, gRNA2: 5′-TCAAGGAAGACAGTGTCTCA) (Synthego Corporation, Redwood City, CA). For genomic depletion of the β3 integrin subunit, one gRNA was designed to induce a frame-shift excision in the coding region of the β3 integrin subunit (gRNA1: 5′GCGCTGGCGGGCGTTGGCGT) (Synthego Corporation, Redwood City, CA). Each gRNA was complexed with Cas9 (Synthego Corporation, Redwood City, CA) in TE buffer for 15 min, with a gRNA: Cas9 ratio of 250:50 pmols. Complexed ribonucleoprotein particles (RNPs) were transfected in PC3-WT cells using a nucleofector 4d X-unit following the manufacturer's guidelines for a 100 µl reaction (Lonza, Basel, Switzerland). After 3 days of recovery, the cells were single-cell sorted using a FACS Aria 2 (BD, Franklin Lakes, NJ) into 96-well plates and allowed to expand for several weeks. Genomic DNA from each clone was isolated, purified, and PCR amplified across the CRISPR target sites and Sanger sequenced. Sanger sequencing deconvolution was carried out using DECODR (DECODR.org) to assess the indel spectrum of each clone. STAT1 genomic depletion resulted in PC3-STAT1KO CRISPR clones referred to as STAT1KO C21 and STAT1KO C1C5. IFIT3 genomic depletion resulted in PC3-IFIT3 CRISPR clones referred to as IFIT3KO C13 and IFIT3KO C15. Finally, for the β3 integrin subunit genomic depletion, one of the clones was unsuccessful, and therefore used as our CRISPR control cells, referred to as PC3-CRISPR control.

### Immunoblot analysis

For immunoblot analysis (IB), the total cell lysates (TCL) and sEV lysates were prepared using radio immuno-precipitation assay (RIPA) buffer (10 mM Tris–HCl, pH 7.4, 150 mM NaCl, 1 mM EDTA, 0.1% SDS, 1% Triton X-100 and 1% sodium deoxycholate) supplemented with protease inhibitors such as calpain, aprotinin, leupeptin, pepstatin, and sodium fluoride. Protein concentration was determined using the Bio-Rad DC^TM^ protein assay kit according to the manufacturer's protocol. Equal amounts of TCL or sEV lysates under reducing (heated with 2-mercaptoethanol) or non-reducing (heated without 2-mercaptoethanol) conditions were separated by sodium dodecyl sulfate-polyacrylamide gel electrophoresis (SDS–PAGE). Proteins were then transferred to a polyvinylidene difluoride (PVDF) membrane (immobilon-E PVDF membrane, pore size 0.45 µm, Millipore). The membrane was incubated with blocking buffer (5% non-fat dry milk in Tris Buffer Saline with 0.1% Tween 20 [TBS-T]) for 1 h at room temperature. The membrane was incubated overnight with respective primary antibodies (Abs) followed by three TBS-T washes of 5 min each at room temperature. To visualize the protein, the membrane was incubated with WesternBright^TM^ ECL (horseradish peroxidase) HRP substrate kits (Advansta Inc., CA, U.S.A.).

### Antibodies

The following Abs were used for IB analyses: mouse monoclonal Abs against the human β6 integrin subunit (6.2A1) [34], CD9 (Santa Cruz, sc-13118), CD63 (Abcam, ab8219), CD81 (Abcam, ab23505), STAT1 (Santa Cruz, sc-271661) and IFIT3 (Santa Cruz, sc-393512); rabbit polyclonal Abs against Actin (Sigma–Aldrich, A2066) and calnexin (Cell signaling, 2433S); rabbit monoclonal TSG101 (Abcam, ab125011); and goat-affinity purified polyclonal Abs against the β6 integrin subunit (R&D Systems, AF2389). The following secondary Abs were used for IB analyses: HRP-linked anti-mouse IgG (Cell Signaling, 7076S), HRP-linked anti-rabbit IgG (Cell Signaling, 7074S) and HRP-linked anti-goat IgG (R&D, HAF019).

### Small extracellular vesicle isolation

sEVs were isolated from serum-free cell culture supernatant of PC3-WT, PC3-CRISPR control, and β6KO C3, β6KO C5, β6KO C7, IFIT3KO C13, IFIT3KO C15, STAT1KO C21 and STAT1KO C1C5 cells, as previously described [44]. Briefly, cells were cultured in 150 mm dishes (ThermoScientific) in RPMI media with l-glutamine supplemented with 10% FBS, and 1% penicillin/streptomycin. Once cells reached 70% confluency, they were placed in starvation media (complete media devoid of FBS) for 48 h after which period sEVs were isolated from culture supernatant collected after a 48 h starvation period. The supernatant was first spun at 2000×***g***, 4°C for 20 min to remove dead cells and debris. The supernatant collected was further spun at 10 000×***g***, 4°C for 35 min in a Beckman Type 45 Ti rotor using a Beckman L8-70 M ultracentrifuge to remove intermediate and large-sized EVs. After the 10 000×***g*** spin, the supernatant was spun at 100 000×***g***, 4°C for 70 min in a Beckman Type 45 Ti rotor using a Beckman L8-70 M ultracentrifuge. The pellet collected was resuspended in PBS and spun a second time to wash at 100 000×***g***, 4°C for 70 min as mentioned above. The final sEV pellet from each cell type was resuspended in 100 µl of PBS and stored at −80°C.

### Iodixanol density gradients isolation

sEVs isolated by differential ultracentrifugation (described above) from PC3-WT, PC3-CRISPR control, β6KO C3, β6KO C5, β6KO C7, IFIT3KO C13, IFIT3KO C15, STAT1KO C21 and STAT1KO C1C5 cells, were further isolated using iodixanol density gradient ultracentrifugation as previously described [44]. Briefly, a 60% stock solution of iodixanol (OptiPrep™, Sigma # 1556) was mixed in a 1 : 1 ratio with a buffer (0.25 M sucrose, 10 mM Tris pH 8.0, and 1 mM EDTA, pH 7.4) to obtain a 30% iodixanol solution. sEVs were mixed with the 30% iodixanol solution and layered at the bottom of an ultracentrifuge SETON tube. Next, 700 µl of 20% iodixanol and 700 µl of 10% iodixanol solutions were layered on top of the 30% iodixanol-sEV suspension to create a discontinuous gradient. Samples were centrifuged for 70 min at 350 000×***g***, 4°C in a SW55Ti rotor using a Beckman L8-70 M ultracentrifuge. A total of 10 consecutive fractions of 260 µl each were collected from top to bottom of the gradient. The refractive index of each fraction was assessed using an ABBE-3 L refractometer, (Fisher Scientific) and their density was calculated. All 10 fractions were diluted with 1 ml of PBS and centrifuged for 70 min at 100 000×***g***, 4°C in a TLA-100.2 rotor using a Beckman, Optima TL ultracentrifuge. The pellets obtained from each of the 10 fractions were washed in 1 ml of PBS, as specified above. The final pellet of all fractions was resuspended in 65 µl of PBS and stored at −80°C.

### Nanoparticle tracking analysis (NTA)

NTA was used to determine the size distribution and concentration of sEVs derived from PC3-WT, PC3-WT cells treated with NS or D2 siRNAs as well as PC3-CRISPR control, β6KO C3, β6KO C5, β6KO C7, IFIT3KO C13, IFIT3KO C15, STAT1KO C21 and STAT1KO C1C5 cells. The samples were loaded in the instrument manually, and the analysis was performed according to the manufacturer's instructions using the NTA software (NS300, Malvern Instruments). The temperature for all experiments was 25°C. sEV suspensions of differential ultracentrifugation-isolated sEVs and iodixanol density gradient-isolated sEV fractions were diluted in PBS at a ratio of 1 : 1000 and/or 1 : 200, respectively. The pooled sEV fractions (2 to 5) were combined and then diluted 1 : 200 in PBS for NTA analysis. The analysis of size distribution and concentration of sEVs was performed under a detection threshold of 5. Video files were captured for a duration of 30 s (repeated three times) with a frame rate of 30 frames per second using the NS300 software version 3.1.54.

### Transmission electron microscopy (TEM)

The pooled iodixanol density gradient-isolated sEV fractions (2 to 5) derived from PC3-CRISPR control and β6KO C5 cells were combined and then diluted 1 : 200 in PBS for TEM analysis as previously described [17]. Briefly, a 5 µl volume of sEVs suspended in 10 mM TRIS-EDTA solution (pH 7.8) was applied to a thin carbon grid that was glow discharged for 2 min using a Pelco Easyglow instrument. A 5 µl volume of freshly made 2% uranyl acetate stain solution was applied and incubated for 2 min on the grid. Excess sample and stain were removed using a Whatman filter paper. The staining process was repeated, and the grid was allowed to dry until imaged. TEM micrographs were collected using a Tecnai T12 TdEM microscope at 100KeV. The images were recorded on a Gatan Oneview 4Kx4K camera. Each image was collected by exposing the sample for 4 s. A total of 100 dose fractionated images were collected into a single micrograph. The imaging data were collected at 1.5 to 2 microns under focus.

### Statistical analysis

The means and standard deviations of size and concentration distributions of sEV preparations measured using NTA derived from PC3-WT, PC3-CRISPR control, β6KO C7, β6KO C3, IFIT3KO C13 or IFIT3KO C15 cells were analyzed. Student's *t*-test was used for comparing two group means. A two-sided *P* value of ≤0.05 is considered statistically significant. Software GraphPad Prism 7 was used for data analysis.

## Results

### IFIT3 and STAT1 protein expression is increased upon down-regulation of the β6 integrin subunit in PrCa cells

**Figure 1.**
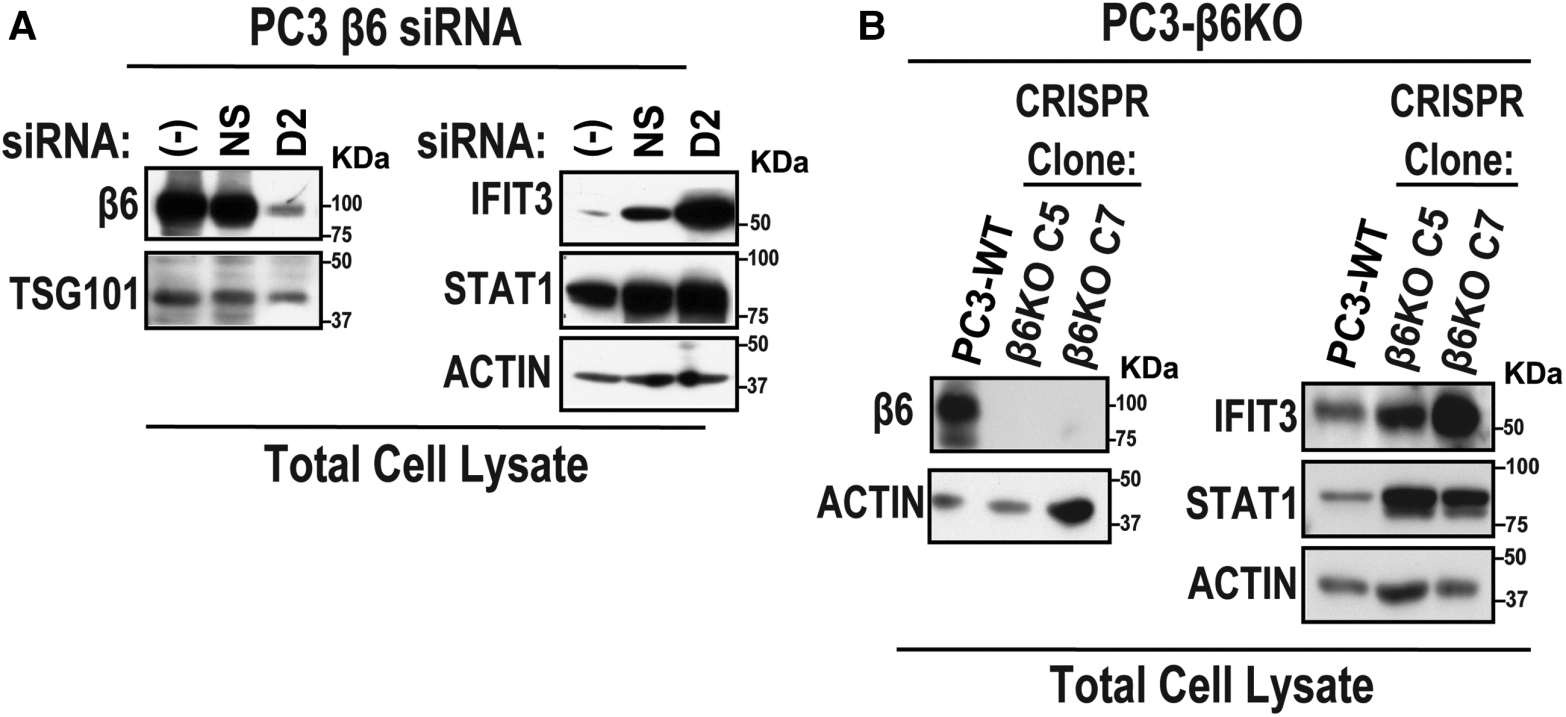
IFIT3 and STAT1 protein expression is increased upon down-regulation of the β6 integrin subunit in PrCa cells. (**A**) Immunoblotting (IB) analysis of total cell lysates (TCL) from PC3 cells containing the β6 integrin subunit, PC3-WT not treated with siRNA (−), PC3 cells treated with non-silencing siRNA (NS), or the β6 integrin subunit siRNA (D2) (30 µg). TCL were separated using 12.5% SDS–PAGE. TCL were examined for the expression of the β6 integrin subunit (left panel, non-reducing SDS–PAGE), IFIT3 and STAT1 (right panel, reducing SDS–PAGE). TSG101 (left panel, non-reducing SDS–PAGE) and Actin (right panel, reducing SDS–PAGE) were used as protein loading controls. (**B**) IB analysis of TCL from PC3-WT cells and PC3-CRISPR clones devoid of the β6 integrin subunit, β6KO C5 and C7 (30 µg). TCL were separated using 12.5% SDS–PAGE. TCL were examined for the expression of the β6 integrin subunit (left panel, non-reducing SDS–PAGE), IFIT3 and STAT1 (right panel, reducing SDS–PAGE). The Actin membrane (right panel, reducing SDS–PAGE) was stripped to visualize IFIT3. Actin (left panel, non-reducing SDS–PAGE), and (right panel, reducing SDS–PAGE) was used as a protein loading control.

We have previously shown that down-regulation of the αvβ6 integrin in PrCa cells results in increased protein expression of STAT1 in total cell lysates (TCL) [24]. We now show that down-regulation of the β6 integrin subunit in PrCa cells also results in elevated IFIT3 protein expression in TCL (Figure 1). We down-regulated the β6 integrin subunit in PC3 wild-type (PC3-WT) cells using a siRNA that specifically targets the β6 integrin subunit (Figure 1A). PC3-WT cells were treated with either non-silencing siRNA (NS) or the β6 integrin subunit siRNA (D2) (Figure 1A, left panel). IFIT3 and STAT1 levels in TCL for each siRNA treatment condition were determined by immunoblotting (IB) analysis (Figure 1A, right panel). We find that compared with the NS siRNA treatment, the levels of IFIT3 and STAT1 are up-regulated upon treatment with the D2 siRNA. TSG101 and Actin were included as loading controls, respectively (Figure 1A, left and right panels). In parallel, to assess the protein levels of IFIT3 and STAT1 in PrCa cells, we used PC3-WT and PC3 cells harboring CRISPR/Cas9-mediated down-regulation of the β6 integrin subunit. CRISPR/Cas9 treatment generated different PC3-β6KO CRISPR clones referred to as β6KO C5 and β6KO C7 (Figure 1B, left panel). As expected, IB analysis of TCL from β6KO C5, β6KO C7 and β6KO C3 cells (Figure 1B, left panel, and data not shown) results in increased protein expression of IFIT3 and STAT1 compared with PC3-WT cells (Figure 1B, right panel). Actin was included as a loading control (Figure 1B, left and right panels).

### IFIT3 is detected in sEVs isolated from PrCa cells devoid of the β6 integrin subunit

Proteomic analysis performed by our group has previously demonstrated that αvβ6 integrin negatively regulates STAT1 and IFIT3 protein levels in both PC3 PrCa cells and their sEVs [24].

**Figure 2.**
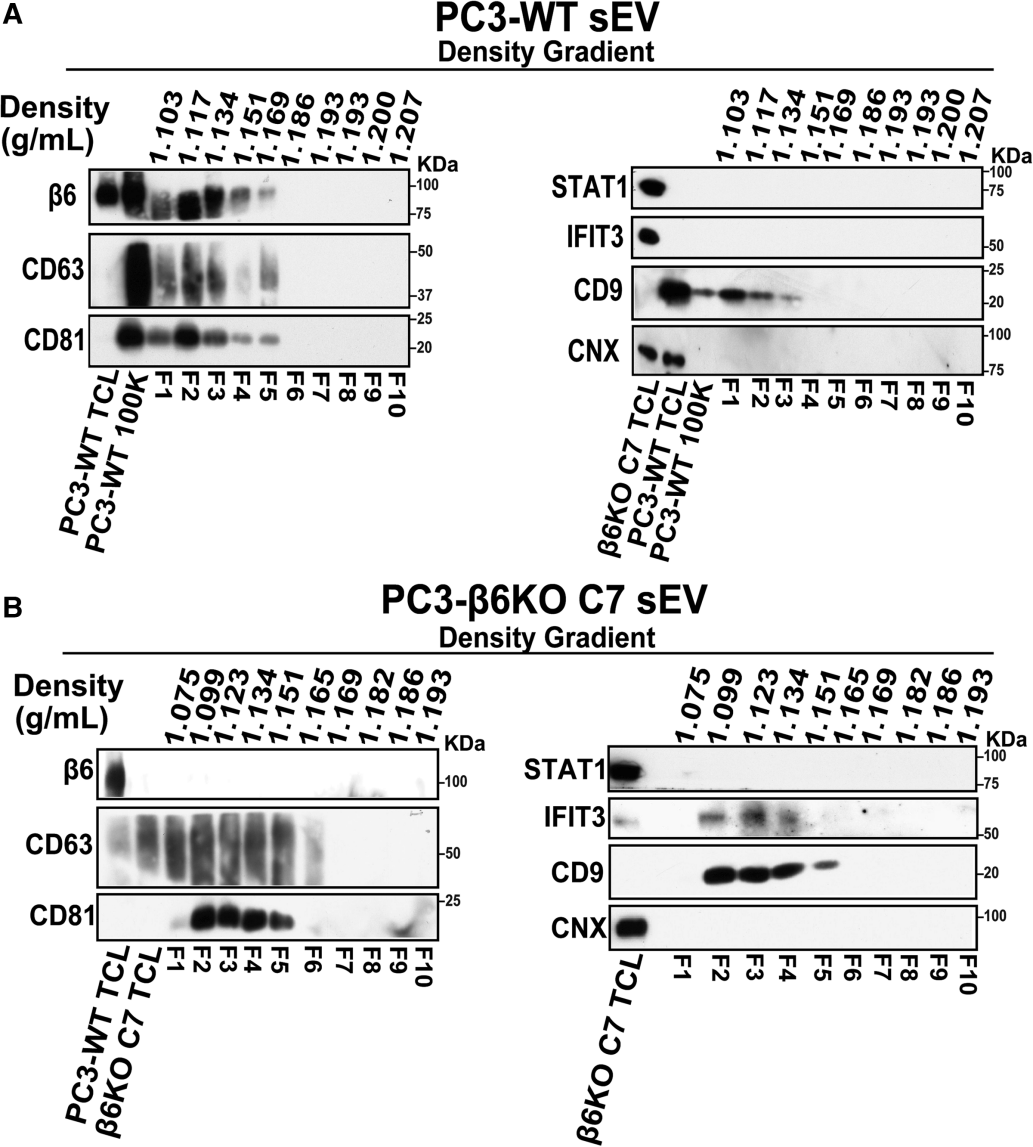
IFIT3 is detected in sEVs isolated via iodixanol density gradients from PrCa cells devoid of the β6 integrin subunit. (**A**) IB analysis of sEV fractions (F1-F10) isolated via iodixanol density gradients and derived from PC3 wild-type (PC3-WT) cells containing the β6 integrin subunit, (PC3-WT). PC3-WT TCL (20 µg), PC3-WT (PC3-WT 100K) sEV lysate (10 µg) and PC3-WT sEV fraction lysates were separated using 12.5% SDS–PAGE. Comparable volumes of each sEV fraction were loaded (30 µl). The 10 consecutive sEV fractions have densities of 1.103, 1.117,1.134,1.151, 1.169, 1.186, 1.193, 1.193, 1.200, and 1.207 g/ml, respectively. Expression of the β6 integrin subunit, CD63, CD81 (left panel, non-reducing SDS–PAGE) as well as STAT1, IFIT3 and CD9 (right panel, reducing SDS–PAGE) was analyzed in sEV fractions 1 to 10. The STAT1 membrane (right panel, reducing SDS–PAGE) was stripped to visualize calnexin (CNX). CNX (right panel, reducing SDS–PAGE) in PC3-β6KO C7, PC3-WT TCL, PC3-WT 100K as well as 10 consecutive PC3-WT derived sEV fractions was analyzed. PC3-WT 100K lysate was the input for the density gradient. (**B**) IB analysis of PC3-β6KO C7 derived sEV fractions isolated via density gradients. Total lysates (TCL 20 µg) from PC3-WT cells containing the β6 integrin subunit, as well as the PC3-CRISPR clone devoid of the β6 integrin subunit, PC3-β6KO C7, and PC3-β6KO C7 sEV fraction lysates were separated using 12.5% SDS–PAGE. Comparable volumes of each sEV fraction were loaded (30 µl). The 10 consecutive sEV fractions have densities of 1.075, 1.099, 1.123, 1.134, 1.151, 1.165, 1.169, 1.182, 1.186, and 1.193 g/ml, respectively. Expression of the β6 integrin, CD63, CD81 (left panel, non-reducing SDS–PAGE), STAT1, IFIT3 and CD9 (right panel, reducing SDS–PAGE) was analyzed in sEV fractions 1 to 10. The STAT1 membrane (right panel, reducing SDS–PAGE) was stripped to visualize calnexin (CNX). CNX (right panel, reducing SDS–PAGE) in the PC3-CRISPR clone lacking the β6 integrin subunit, PC3-β6KO C7 TCL and PC3-β6KO C7 derived sEV fractions was analyzed. A and B, TCL = total cell lysates.

We first assessed the protein levels of IFIT3 in 10 consecutive sEV fractions derived from the β6 integrin subunit expressing PC3-WT cells and from β6KO C7 cells that lack the β6 integrin subunit (Figure 2) by using IB analysis. As expected from previous studies [17], we find that the β6 integrin subunit is expressed in PC3-WT sEV fractions 1 to 5 which have a density range of 1.103–1.169 g/ml (Figure 2A, left panel). The tetraspanins, CD63 and CD81, which are known sEV markers [19], are also expressed in the same fractions containing the β6 integrin subunit (Figure 2A, left panel). Not surprisingly, we find that IFIT3 and STAT1 protein expression is undetectable from PC3-WT sEV fractions (Figure 2A, right panel). Moreover, the tetraspanin CD9, a canonical sEV marker [19], is expressed in fractions 1 to 3, which collectively have a density range of 1.103–1.134 g/ml (Figure 2A, right panel). The endoplasmic reticulum marker (ER), calnexin (CNX) [45], is undetectable from all the sEV fractions, but present in PC3-WT and β6KO C7 TCL (Figure 2A, right panel). CNX absence excludes the possibility of contamination of ER proteins in the isolated sEVs [19]. These data demonstrate that the presence of the β6 integrin subunit in PrCa cells, prevents IFIT3 expression or localization in PrCa-derived sEVs.

In the sEV fractions derived from β6KO C7 cells, we confirm the absence of the β6 integrin subunit (Figure 2B, left panel). Meanwhile, the sEV markers CD63 as well as CD81 are found to be expressed in sEV fractions 2 to 5 which cover a density range of 1.099–1.151 g/ml (Figure 2B, left panel). In the absence of the β6 integrin subunit, IFIT3 is expressed in sEV fractions 2 to 4, which is also a reproducible finding in β6KO C3 cells (Figure 2B, right panel, and data not shown). However, STAT1 expression is not detected in any of the sEV fractions derived from β6KO C7 cells (Figure 2B, right panel) where, as expected we find that CD9 is expressed in sEV fractions 2 to 5 (Figure 2B, right panel). The absence of CNX in the sEV fractions confirms the lack of ER proteins in the isolated sEVs [19] (Figure 2B, right panel). These results further establish that the αvβ6 integrin plays a role in down-regulating IFIT3 protein expression in PrCa cell-derived sEVs.

**Figure 3.**
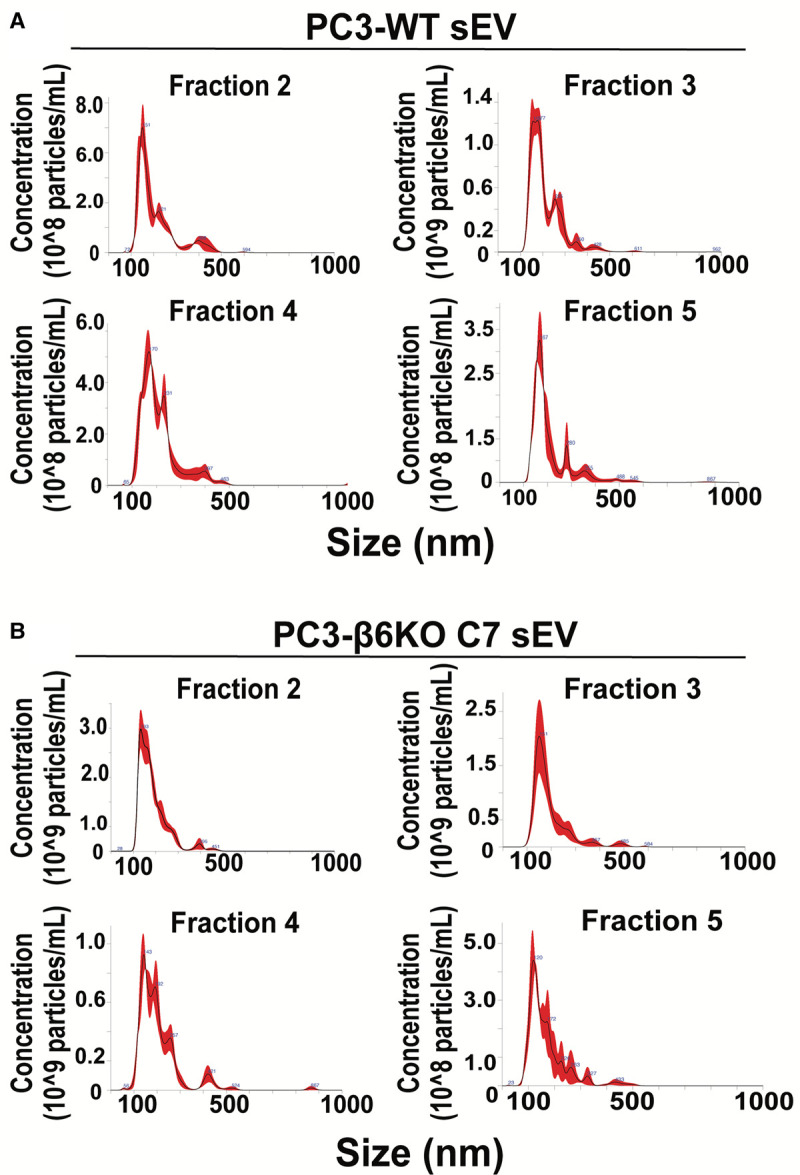
Characterization of the size distribution of density gradient-isolated sEV fractions devoid of the β6 integrin subunit. (**A**) Nanoparticle tracking analysis (NTA) measurement of size distribution and concentration of sEV fractions 2 to 5 derived from cells containing the β6 integrin subunit (PC3-WT). (**B**) NTA measurement of size distribution and concentration of sEV fractions 2 to 5 derived from the PC3-CRISPR clone devoid of the β6 integrin subunit, PC3-β6KO C7.

Next, we assessed the size and concentration of sEV fractions using nanoparticle tracking analysis (NTA). We isolated sEVs from PC3-WT or β6KO C7 cells by differential ultracentrifugation (100 000×***g***) [44], which are referred to as PC3-WT 100K or β6KO C7 100K. Iodixanol density gradients were used to isolate sEV fractions from PC3-WT 100K or β6KO C7 100K sEVs and remove contaminants by separating the sEVs on different buoyant densities. We collected 10 sEV fractions from top to bottom of the iodixanol density gradient. We then proceeded to assess the size and concentration of sEV fractions derived from PC3-WT or β6KO C7 cells (Figure 3). The mean sizes of sEV fractions 2 to 5 derived from PC3-WT are 205.6 nm, 209.4 nm, 211.4 nm, and 207.5 nm, respectively (Figure 3A). The mean sizes of sEV fractions 2 to 5 derived from β6KO C7 cells are 185.1 nm, 191.2 nm, 204.1 nm and 167.2 nm, respectively (Figure 3B). These results align with our previous research findings where we determined that sEVs derived from PrCa cells are less than 200 nm in size [16,46]. Furthermore, we did not detect significant concentration changes in sEV preparations derived from the β6 integrin subunit-expressing PC3-WT cells (*n* = 2) compared with sEV fractions derived from the PC3-CRISPR clones devoid of the β6 integrin subunit, β6KO C7 (*n* = 2) or β6KO C3 (*n* = 2, data not shown). Specifically, the mean concentration of two sEV preparations that comprise fractions 2 to 5 derived from PC3-WT, β6KO C7 or β6KO C3 is 6.51 × 10^8^, 1.28 × 10^9^, or 7.99 × 10^8^ particles/ml, respectively (Figure 3A,B). Similar results were obtained for sEVs isolated via differential ultracentrifugation (100 000×***g***) from the PC3-CRISPR control cells expressing the β6 integrin subunit, or PC3-β6KO CRISPR clones (Supplementary Figure S1).

### Down-regulation of the β6 integrin subunit in PrCa cells does not affect sEV size distribution

**Figure 4.**
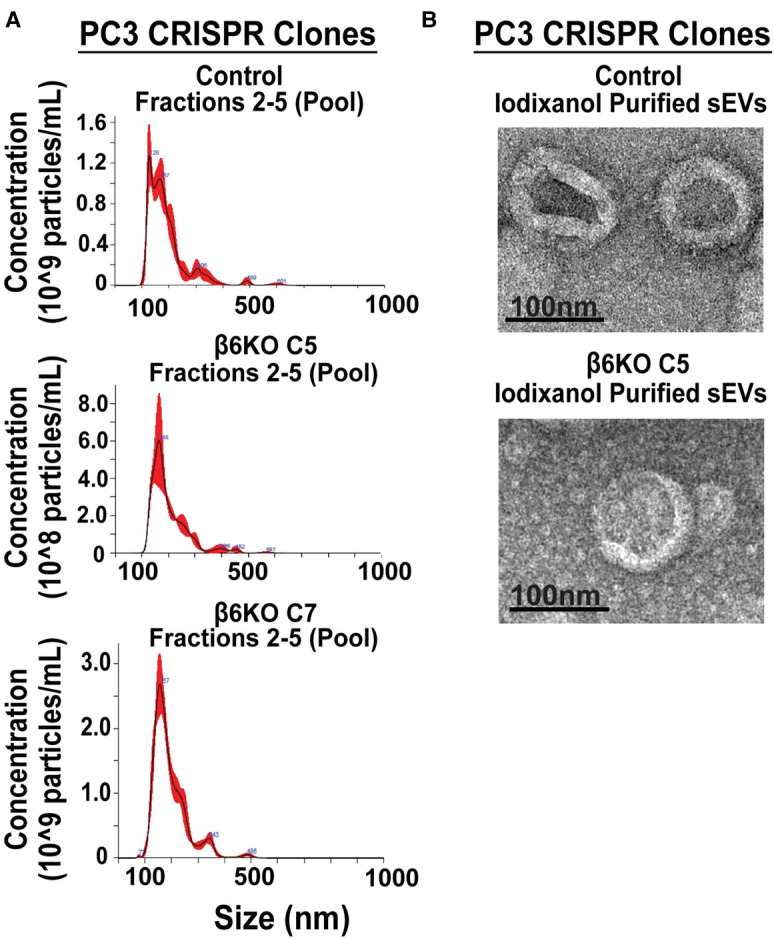
Down-regulation of the β6 integrin subunit in PrCa cells does not affect size distribution of density gradient-isolated sEVs. (**A**) NTA measurement of size distribution and concentration of density gradient-isolated sEVs (pooled fractions 2 to 5) derived from cells containing the β6 integrin subunit, PC3-CRISPR control and the PC3-CRISPR clones devoid of the β6 integrin subunit, β6KO C5 and β6KO C7. (**B**) Transmission electron microscopy analysis of density gradient-isolated sEVs (pooled fractions 2 to 5) derived from PC3-CRISPR control and β6KO C5 cells. Scale bar = 100 nm.

It has been established that sEVs exhibit a size range of 50–200 nm [47]. Therefore, we wanted to assess whether the absence of the β6 integrin subunit had any impact on sEV size. To answer this question, we assessed the sEV size by NTA. Using the PC3-CRISPR control cells that express the β6 integrin subunit or the PC3-CRISPR clones devoid of the β6 integrin subunit β6KO C5 and β6KO C7, we isolated sEVs via density gradients and pooled sEV fractions 2 to 5. NTA analysis shows that the mean sizes of pooled sEV fractions 2 to 5 derived from the PC3-CRISPR control, β6KO C5, and β6KO C7 cells are 188.8 nm, 165.5 nm, and 193.3 nm, respectively (Figure 4A). Transmission electron microscopy (TEM) analysis displays the size and morphology of density gradient-isolated sEVs derived from the PC3-CRISPR control and β6KO C5 cells (Figure 4B). According to the TEM analysis of sEV fractions derived from PC3-CRISPR control cells, 76% of the sEVs are <100 nm and 24% are between 100 nm and 200 nm in size. Specifically, out of 41 sEVs derived from PC3-CRISPR control cells, 31 had a size <100 nm and 10 had a size between 100 nm and 200 nm. For sEV fractions derived from β6KO C5 cells, 82% of the sEVs are <100 nm and 18% are between 100 nm and 200 nm in size. Specifically, for sEVs derived from β6KO C5 cells, we counted a total of 55 sEVs in which 45 are <100 nm in size and 10 are between 100 nm and 200 nm in size. As a result, we concluded that there is no significant size difference between sEVs containing or devoid of the β6 integrin subunit.

### IFIT3 down-regulation results in increased STAT1 levels in PrCa cell-derived sEVs

**Figure 5.**
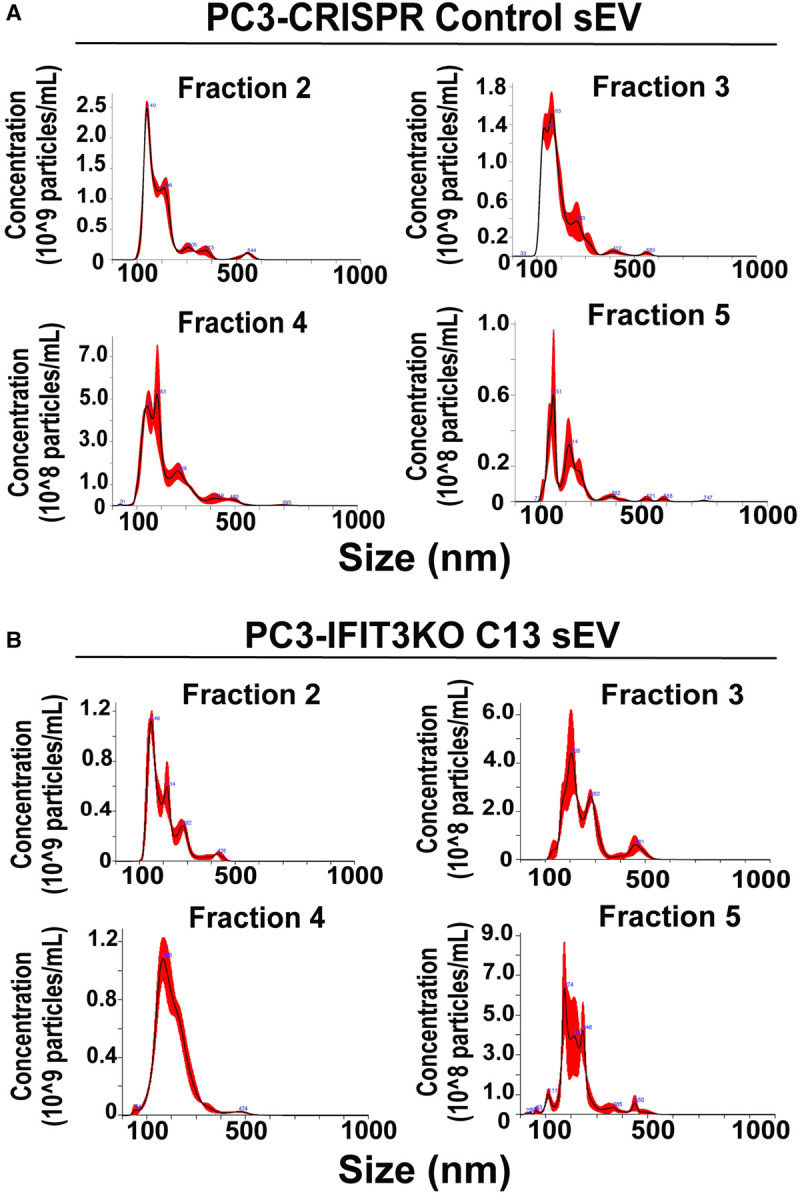
Characterization of density gradient-isolated sEVs derived from PC3-CRISPR control and IFIT3KO PrCa cells. (**A**) NTA measurement of size distribution and concentration of PC3-CRISPR control cell-derived sEV fractions 2 to 5. (**B**) NTA measurement of size distribution and concentration of sEV fractions 2 to 5 derived from a representative PC3-CRISPR clone devoid of IFIT3 (PC3-IFIT3KO C13).

It has been previously shown that STAT1 regulates IFIT3 expression, as STAT1 is a key protein needed for relaying the transcriptional activity of the ISG proteins [38]. However, whether IFIT3 has any regulatory effect on STAT1 expression is not understood. To analyze this potential interaction, we utilized PrCa cells harboring CRISPR/Cas9-mediated down-regulation of IFIT3 (IFIT3KO clone C13 and IFIT3KO clone C15) as well as PC3-CRISPR control cells. First, we isolated sEVs from IFIT3KO C13 and PC3-CRISPR control cells via differential ultracentrifugation, designated as IFIT3KO C13 100K and PC3-CRISPR control 100K sEVs. After this step, we isolated IFIT3KO C13 100K and PC3-CRISPR control 100K sEVs using density gradients and characterized the sEV size distribution by NTA (Figure 5). We find that the mean sizes of individual sEV fractions 2 to 5 derived from PC3-CRISPR control cells are 197.8 nm, 189.7 nm, 212.7 nm, and 208.0 nm, respectively (Figure 5A). Furthermore, IFIT3KO C13 sEV fractions 2 to 5 have mean sizes of 201.1 nm, 251.1 nm, 201.5 nm, and 219.6 nm, respectively (Figure 5B). These results show that the sEVs isolated from PC3-CRISPR control or IFIT3KO C13 cells fall within the reported sEV size range [47] and are not affected by CRISPR-based gene editing.

We did not detect significant concentration changes in sEV preparations derived from the PC3-CRISPR control cells (*n* = 2) when compared with sEV fractions derived from the PC3-CRISPR clones devoid of IFIT3, IFIT3KO C13 (*n* = 2), and IFIT3KO C15 (*n* = 2, data not shown). Specifically, the mean concentrations of two sEV preparations that comprise fractions 2 to 5 derived from PC3-CRISPR control, IFIT3KO C13 or IFIT3KO C15 cells are 1.10 × 10^9^, 9.2 × 10^8^, or 1.34 × 10^9^ particles/ml, respectively (Figure 5A,B). Thus, we show that IFIT3 down-regulation does not affect sEV release.

**Figure 6.**
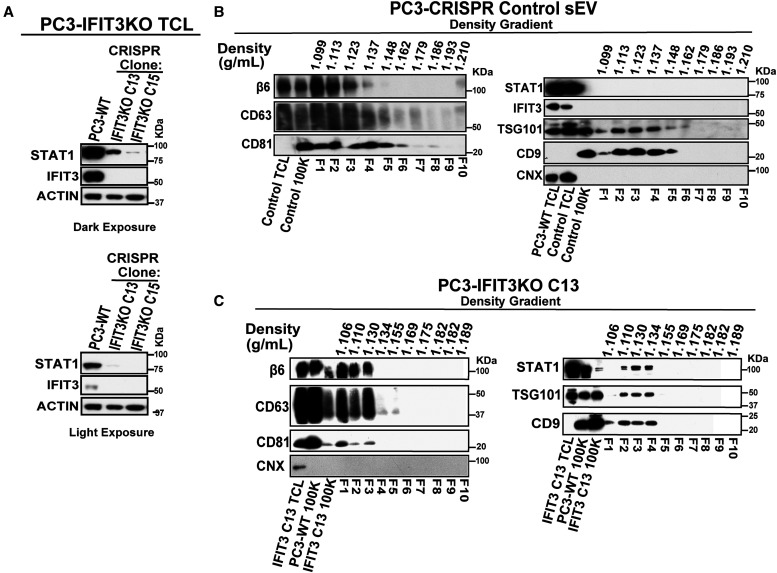
IFIT3 down-regulation results in increased STAT1 levels in PrCa cell-derived sEVs. (**A**) IB analysis of PC3-WT and the PC3-CRISPR clones devoid of IFIT3: PC3-IFIT3KO C13 and PC3-IFIT3KO C15 TCL (total cell lysates) (30 µg). TCL were separated using 12.5% SDS–PAGE and examined for the expression of STAT1 and IFIT3 (reducing SDS–PAGE). The IFIT3 membrane (reducing SDS–PAGE) was stripped to visualize Actin. Actin (reducing SDS–PAGE) was used as a protein loading control. Dark and light exposures shown (Top and bottom, respectively). (**B**) IB analysis of cells expressing the β6 integrin subunit, PC3-CRISPR control cell-derived sEV fractions isolated via density gradients. PC3-CRISPR control TCL (20 µg), PC3-CRISPR control (CRISPR control 100K) sEV lysate (10 µg), and PC3-CRISPR control sEV fraction lysates were separated by using 12.5% SDS–PAGE. Comparable volumes of each sEV fraction were loaded (30 µl). The 10 consecutive sEV fractions have a density of 1.099, 1.113,1.123,1.137, 1.148, 1.162, 1.179, 1.186, 1.193, and 1.210 g/ml, respectively. Expression of the β6 integrin subunit, CD63, CD81 (left panel, non-reducing SDS–PAGE), STAT1, IFIT3, TSG101 and CD9 (right panel, reducing SDS–PAGE) was analyzed in sEV fractions 1 to 10. IFIT3 (right panel, reducing SDS–PAGE) and STAT1 membranes (right panel, reducing SDS–PAGE) were stripped to visualize TSG101 and calnexin (CNX), respectively. CNX expression (right panel, reducing SDS–PAGE) in PC3-CRISPR control TCL, CRISPR control 100K as well as 10 consecutive PC3-CRISPR control cell-derived sEV fractions is shown. CRISPR control 100K sEV lysate was used as input for the density gradient. (**C**) IB analysis of sEV fractions derived from a PC3-CRISPR clone devoid of IFIT3, designated as PC3-IFIT3KO C13, and isolated via density gradients. PC3-IFIT3KO C13 TCL (20 µg), PC3-IFIT3KO C13 (IFIT3KO C13 100K) sEV lysate (10 µg) and PC3-IFIT3KO C13 sEV fraction lysates were separated using 12.5% SDS–PAGE. Comparable volumes of each sEV fraction were loaded (30 µl). The 10 consecutive sEV fractions have a density of 1.106, 1.110,1.130,1.134, 1.155, 1.169, 1.175, 1.182, 1.182, and 1.189 g/ml, respectively. Expression of the β6 integrin subunit, CD63 and CD81 (left panel, non-reducing SDS–PAGE) is analyzed in sEV fractions 1 to 10. The β6 integrin subunit membrane (left panel, reducing SDS–PAGE) was stripped to visualize calnexin (CNX). CNX expression (left panel, non-reducing SDS–PAGE) in PC3-IFIT3KO C13 TCL, PC3-WT 100K, IFIT3 C13 100K and 10 consecutive PC3-IFIT3KO C13 cell-derived sEV fractions is shown. Expression of STAT1, TSG101 and CD9 (right panel, reducing SDS–PAGE) is analyzed. IFIT3KO C13 100K was the input for the density gradient.

We then proceeded to analyze the content of 10 consecutive sEV fractions derived from the PC3-CRISPR control cells and the PC3-CRISPR clones devoid of IFIT3 (IFIT3KO C13 and IFIT3KO C15). IB analysis of IFIT3KO C13 and IFIT3KO C15 TCL confirms the absence of IFIT3 (Figure 6A). Moreover, STAT1 expression is decreased in IFIT3KO C13, and IFIT3KO C15 cells compared with PC3-WT cells (Figure 6A). Actin was used as a loading control (Figure 6A). PC3-CRISPR control cell-derived sEV fractions 1 to 4, which have a density range of 1.099–1.137 g/ml, contain the β6 integrin subunit as well as the sEV markers CD63 and CD81 (Figure 6B, left panel). Furthermore, IFIT3 as well as STAT1 expression is undetectable from all sEV fractions (Figure 6B, right panel). The sEV markers TSG101 and CD9 are specifically detected in fractions 2 to 5 corresponding to a density range of 1.113–1.148 g/ml (Figure 6B, right panel). CNX absence in all 10 sEV fractions confirms the lack of contamination of ER proteins in our isolated sEVs (Figure 6B, right panel).

In sEV fractions derived from the PC3-CRISPR clone devoid of IFIT3, termed IFIT3KO C13, the β6 integrin subunit as well as CD63 and CD81 are abundant in fractions 1 to 3, which span the density range of 1.106–1.130 g/ml (Figure 6C, left panel). CNX is undetectable from all 10 sEV fractions, but present in IFIT3KO C13 TCL (Figure 6C, left panel). Furthermore, STAT1 expression is clearly observed in sEV fractions 2 to 4 derived from IFIT3KO C13 and IFIT3KO C15 cells (Figure 6C, left panel and data not shown), which cover a density range of 1.110–1.134 g/ml (Figure 6C, right panel). The levels of TSG101 and CD9 are enriched in the sEV fractions compared with IFIT3KO C13 TCL (Figure 6C, right panel). These results suggest that there is an interaction in which STAT1 compensates for the loss of IFIT3. Therefore, our results show that STAT1 is increased in sEVs derived from PrCa cells devoid of IFIT3.

### STAT1 down-regulation affects IFIT3 expression in PrCa cells and their sEVs

**Figure 7.**
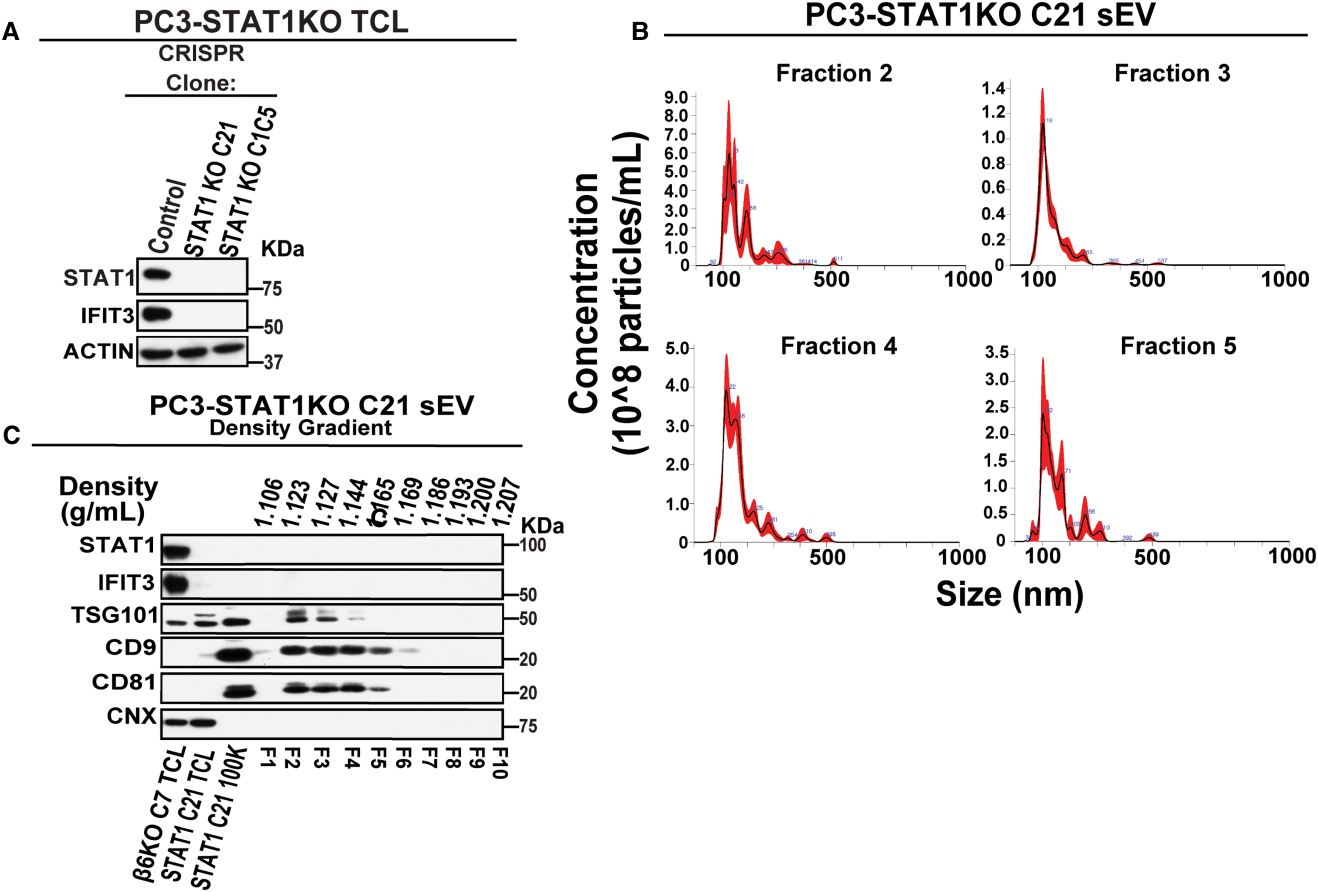
STAT1 down-regulation affects IFIT3 protein expression in PrCa cells and their sEVs. (**A**) IB analysis of PC3-CRISPR control cells and PC3-CRISPR clones devoid of STAT1, PC3-STAT1KO C21 as well as PC3-STAT1KO C1C5 TCL (total cell lysates) (30 µg). TCL were separated using 12.5% SDS–PAGE. TCL were examined for the expression of STAT1, IFIT3, and Actin (reducing SDS–PAGE). The IFIT3 membrane (reducing SDS–PAGE) was stripped to visualize Actin. Actin was used as a protein loading control. (**B**) NTA measurement of size distribution and concentration of PC3-STAT1KO C21 derived sEV fractions 2-5. (**C**) IB analysis of sEV fractions derived from a PC3-CRISPR clone lacking STAT1, designated as PC3-STAT1KO C21, and isolated via density gradients. PC3-β6KO C7, PC3-STAT1KO C21 TCL (20 µg), PC3-STAT1KO C21 (STAT1KO C21 100K) sEV lysate (10 µg) and PC3-STAT1KO C21 sEV fraction lysates were separated by using 12.5% SDS–PAGE. Comparable volumes of each sEV fraction were loaded (30 µl). The 10 consecutive sEV fractions have a density of 1.106, 1.123,1.127,1.144 1.165, 1.169,1.186, 1.193, 1.200, and 1.207 g/ml, respectively. Expression of STAT1, IFIT3, TSG101, CD9 and CD81 (reducing SDS–PAGE) was analyzed in sEV fractions 1 to 10. The IFIT3, STAT1 and CD9 membranes (reducing SDS–PAGE) were stripped to visualize TSG101, calnexin (CNX), and CD81 respectively. CNX expression (reducing SDS–PAGE) was analyzed in PC3-CRISPR clone devoid of the β6 integrin subunit, PC3-β6KO C7 as well as PC3-CRISPR clone lacking STAT1, PC3-STAT1KO C21 TCL, STAT1KO C21 100K and 10 consecutive PC3-STAT1KO C21 derived sEV fractions. PC3-STAT1KO C21 100K sEV lysate was used as input for the density gradient.

Our results prompted us to assess IFIT3 protein expression levels in PrCa-derived sEVs in the absence of STAT1. For this purpose, we used PrCa cells harboring CRISPR/Cas9-mediated down-regulation of STAT1, thus generating STAT1KO C21 and STAT1KO C1C5 CRISPR clones. We first proceeded to isolate sEVs from STAT1KO C21 cells via differential ultracentrifugation (STAT1KO C21 100K). Then, we isolated sEVs from STAT1KO C21 cells using density gradients. IB analysis of TCL from PC3-CRISPR control cells and the PC3-CRISPR clones devoid of STAT1, STAT1KO C21 and STAT1KO C1C5, confirms the lack of STAT1 expression in STAT1KO clones compared with PC3-CRISPR control cells (Figure 7A). Noteworthy, IFIT3 expression is undetectable from both STAT1KO C21 and STAT1KO C1C5 cells (Figure 7A). These results are consistent with previous studies that posit STAT1 as a key player in regulating the expression of the ISG proteins such as IFIT3 [37]. Using NTA analysis, we characterized the size distribution of density gradient-isolated sEVs derived from STAT1KO C21 cells (Figure 7B). The mean sizes of sEV fractions 2 to 5 derived from STAT1KO C21 cells are 168.3 nm, 152.8 nm, 170.9 nm, and 154.5 nm, respectively. Furthermore, we did not detect significant concentration changes in sEV preparations derived from the PC3-CRISPR control cells (Figure 5A) when compared with sEV preparations derived from the PC3-CRISPR clones devoid of STAT1, STAT1KO C21 or STAT1KO C1C5 (Figure 7B). Thus, we show that STAT1 down-regulation does not affect sEV release.

We then proceeded to analyze by IB 10 consecutive sEV fractions derived from STAT1KO C21 cells. According to the IB analysis, STAT1 and IFIT3 are undetectable from all sEV fractions derived from the STAT1KO C21 and STAT1KO C1C5 cells (Figure 7C and, data not shown). The sEV markers TSG101, CD9 and CD81 are abundant in sEV fractions 2 to 4, which collectively represent a density range of 1.123–1.144 g/ml. CNX is undetectable from all 10 sEV fractions, but expressed in β6KO C7 and STAT1KO C21 TCL. These data establish that knocking down STAT1 in PrCa cells results in IFIT3 down-regulation in STAT1KO cell-derived sEV fractions. Our results further show that STAT1 down-regulation causes the ablation of IFIT3 expression in PrCa cells and their sEVs.

## Discussion

Our study reveals that down-regulation of the β6 integrin subunit via siRNA (D2) or CRISPR/Cas9 techniques in PrCa cells results in increased protein expression of IFIT3 and STAT1 in PrCa cells. IFIT3 expression, but not STAT1, is increased in density gradient-isolated sEVs derived from PrCa cells devoid of the β6 integrin subunit. We demonstrate that loss of IFIT3 generates sEVs enriched in STAT1, but reduces the levels of STAT1 in the cells. As expected, IFIT3 is not detectable in STAT1 negative cells or sEVs. Overall, these results demonstrate for the first time that IFIT3 plays a novel role in regulating STAT1 expression in PrCa cells and their sEVs. We propose that STAT1 enrichment in PrCa-derived sEVs devoid of IFIT3 is a compensatory mechanism for IFIT3 loss.

Previously, our group has shown the relevance of the αvβ6 integrin in cancer progression by establishing that this integrin can be transferred from PrCa cells to recipient cells via sEVs [16,17]. We have also demonstrated that the αvβ6 integrin contained in PrCa cell-derived sEVs plays an unequivocal role in promoting cell proliferation, survival, and angiogenesis [16,25,26]. Moreover, proteomic analysis previously published by our group, shows that the αvβ6 integrin plays a role in down-regulating the protein expression of different ISG such as IFIT3, in PrCa cells as well as in the sEVs released by these cells [24]. Results from our current study align with these findings by showing that IFIT3 is expressed in sEVs derived from PrCa cells devoid of the β6 integrin subunit whereas IFIT3 is undetectable from sEVs derived from PrCa cells that contain the β6 integrin subunit.

Aside from its antiviral activity, the role of IFIT3 in cancer is poorly understood, and even less so in PrCa. A study conducted by Weichselbaum and coworkers showed that IFIT3 is down-regulated in PrCa human tumors [48]. However, IFIT3 expression has been shown to be detrimental for patient outcomes in other cancers. In oral squamous cell carcinoma (OSCC), for instance, IFIT3 ectopic expression has been shown to correlate with poor survival in OSCC patients [39]. In pancreatic ductal carcinoma (PDAC), IFIT3 expression was proposed to be a prognostic marker of PDAC, as well as shown to mediate antiapoptotic activity and chemoresistance of PDAC cells [49]. Notwithstanding, our findings are consistent with other research supporting an anti-proliferative role of IFIT3 in cancer [42], as we demonstrate a new role for the αvβ6 integrin as a negative regulator of IFIT3. For example, in pro-monocytic myeloid leukemia cells, IFIT3 expression induced G1 phase arrest by increasing the expression of the cyclin-dependent kinase inhibitors, p21 and p27 [37]. In another study, it was shown that IFIT3 overexpression in lung cancer cells was able to exert an anti-tumor activity [50]. In fact, IFIT3 overexpression caused the down-regulation of NFκB pathways, cyclin D1, c-Myc, and PCNA. IFIT3 has also been shown to reduce lung cancer cell proliferation, migration, and epithelial mesenchymal transition [42]. The same study demonstrated that IFIT3 tumor-suppressor function was dependent on increased expression of p53. Moreover recently, Wang et al. [51] showed that the expression levels of IFIT3 in the peripheral blood from patients with acute promyelocytic leukemia is low compared with healthy controls. Therefore, it is conceivable that the αvβ6 integrin down-regulates IFIT3 as a mechanism to promote tumor growth.

We also show for the first time that STAT1 is detected in sEVs derived from PrCa cells devoid of IFIT3 and isolated via density gradients. Noteworthy, Cossetti et al., detected STAT1 in density gradient-isolated sEVs from neural stem/precursor cells only by prior cell-stimulation with pro-inflammatory cytokine cocktails [52]. In our results, however, we observe STAT1 expression in sEVs without exposing our cells to pro-inflammatory cytokines and more importantly, in the absence of IFIT3. Abundant STAT1 expression in PrCa-derived sEVs devoid of IFIT3 in the absence of cytokine stimulation, might be a cell line-specific effect, since neural stem/precursor cells might require higher levels of stimulation to induce ISG proteins compared with PrCa cells. Overall, these results suggest that there is a compensatory mechanism in which increased expression of STAT1 counterbalances the loss of IFIT3. Moreover, STAT1 is known to have a tumor suppressor role [31–33] whereas IFIT3 has been shown to play a dual role in cancer [39,42]. Therefore, we speculate that the relationship between STAT1 and IFIT3 in this study is a mechanism of balance between proliferative and anti-proliferative role of IFIT3 in a context-dependent manner.

**Figure 8.**
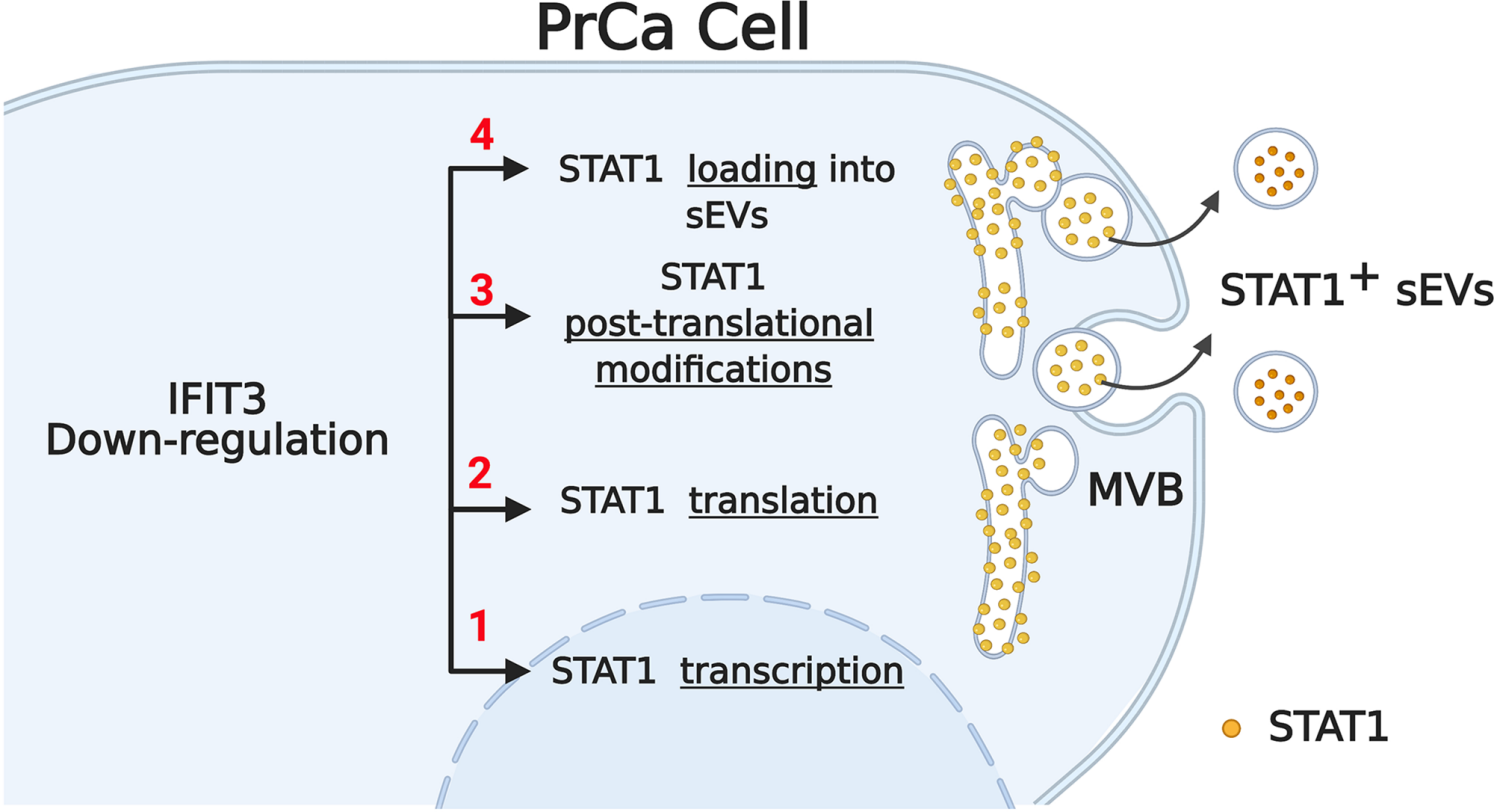
Proposed model for IFIT3-mediated regulation of STAT1 expression and increased loading into sEVs. For our proposed model in PrCa cells, we speculate that increased expression of STAT1 in sEVs mediated by IFIT3 down-regulation is potentially occurring through any of the following mechanisms. Increased expression of STAT1 in sEVs may be a consequence of changes at the (1) transcriptional, (2) translational, or (3) post-translational level. As a consequence of these modifications, (4) increased STAT1 loading into multivesicular bodies (MVBs) results in STAT1 enrichment in sEVs released by PrCa cells. Created with Biorender software.

In the literature, it is well known that STAT1 induces the expression of IFIT proteins by serving as a component of the heterotrimeric ISGF3 transcription complex [38]. Other research has shown that IFIT3 associates with STAT1 and STAT2 to mediate their interaction, heterodimerization, and enhance STAT1 and STAT2 nuclear translocation [41]. However, no other studies have presented evidence of IFIT3 negative regulation of STAT1 expression and increased loading into sEVs. The mechanism by which IFIT3 regulates STAT1 in PrCa needs to be further explored. For our proposed model (Figure 8), we speculate that expression of STAT1 in sEVs mediated by IFIT3 down-regulation is potentially occurring through any of the following mechanisms: (1) transcriptional, (2) translational, or (3) post-translational levels. As a consequence of these modifications, (4) increased STAT1 loading into multivesicular bodies (MVBs) results in STAT1 enrichment in sEVs released by PrCa cells.

As sEVs play a pivotal role in cell–cell communication and have the potential to alter recipient cells’ phenotype and function [23–25], we therefore believe that PrCa-derived sEVs containing the proteins STAT1 or IFIT3, have an important physiological relevance. STAT1 for instance, is known to mediate macrophage M1 polarization [53,54]. Along the same lines, IFIT3 has been described as a novel marker of M1 macrophages [55]. Therefore, we foresee that transfer of sEVs containing IFIT3 or STAT1 to other cells in the TME, such as macrophages, might change their surface receptor expression as well as their cytokine release into a M1 pro-inflammatory response.

In summary, our results demonstrate that loss of IFIT3 generates sEVs enriched in STAT1. We thus propose that the observed STAT1 enrichment in sEVs is a compensatory mechanism for the loss of IFIT3. These results open new promising avenues to explore the intrinsic role of PrCa-derived sEVs containing IFIT3 or STAT1, and how transfer of these cargoes to neighboring non-cancerous cells affects their phenotype as well as their function.

## Data Availability

All of the primary data that is presented in this study can be requested in electronic form by contacting Dr. Lucia Languino (lucia.languino@jefferson.edu).

## Abbreviations

CNX: calnexin
ECL: enhanced chemiluminescence
ER: endoplasmic reticulum
HRP: horseradish peroxidase
IFIT3: interferon-induced protein with tetratricopeptide repeat 3
ILVs: intraluminal vesicles
ISG: interferon-stimulated gene
MVEs: multivesicular endosomes
NTA: Nanoparticle tracking analysis
PrCa: Prostate cancer
sEVs: small extracellular vesicles
STAT1: signal transducer activator of transcription 1
T-BST: Tris-buffered saline Tween
TCL: total cell lysates
TEM: Transmission electron microscopy
