# Supplementary material for: IFIT3 (interferon induced protein with tetratricopeptide repeats 3) modulates STAT1 expression in small extracellular vesicles
Source: Biochem J. 2021 Nov 9;478(21):3905–21. doi: 10.1042/BCJ20210580 (PMC9121857; doi:10.1042/BCJ20210580)
Supplement: Supplementary Figure S1 [file BCJ-478-3905-s1.pdf]

Supplementary Figure S1

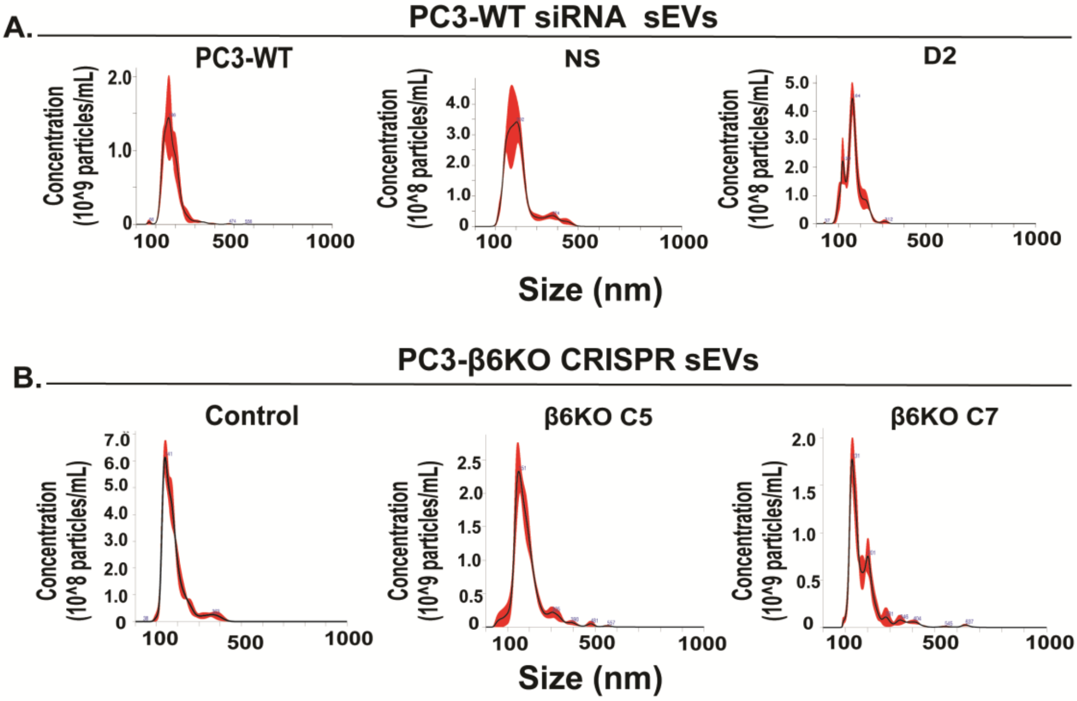

Supplementary Figure S1. Downregulation of the β6 integrin subunit in PrCa cells does not affect size distribution of sEVs isolated via differential ultracentrifugation. **(A)** NTA analysis of the size distribution and concentration of sEVs isolated via differential 100,000 x g ultracentrifugation. PC3-WT siRNA sEVs were derived from PC3-WT transfected with non-silencing siRNA (NS) or the β6 integrin subunit siRNA (D2). **(B)** NTA analysis of the size distribution and concentration of sEVs isolated via differential 100,000 x g ultracentrifugation. sEVs were derived from the PC3-CRISPR clone containing the β6 integrin subunit, PC3-CRISPR control cells as well as PC3-CRISPR clones devoid of the β6 integrin subunit, PC3-β6KO C5 and PC3-β6KO C7.
